# RFCM-PALM: In-Silico Prediction of S-Palmitoylation Sites in the Synaptic Proteins for Male/Female Mouse Data

**DOI:** 10.3390/ijms22189901

**Published:** 2021-09-14

**Authors:** Soumyendu Sekhar Bandyopadhyay, Anup Kumar Halder, Monika Zaręba-Kozioł, Anna Bartkowiak-Kaczmarek, Aviinandaan Dutta, Piyali Chatterjee, Mita Nasipuri, Tomasz Wójtowicz, Jakub Wlodarczyk, Subhadip Basu

**Affiliations:** 1 Department of Computer Science and Engineering, Jadvapur University, Kolkata 700032, India; soumyabane@gmail.com (S.S.B.); anup21.halder@gmail.com (A.K.H.); aviinandaandutta@gmail.com (A.D.); mitanasipuri@gmail.com (M.N.); 2 Department of Computer Science and Engineering, School of Engineering and Technology, Adamas University, Barasat, Kolkata 700126, India; 3 Department of Computer Science and Engineering, University of Engineering & Management, Kolkata 700156, India; 4 The Nencki Institute of Experimental Biology, Polish Academy of Sciences, 3 Pasteur Street, 02-093 Warsaw, Poland; m.zareba-koziol@nencki.edu.pl (M.Z.-K.); a.bartkowiak@nencki.edu.pl (A.B.-K.); t.wojtowicz@nencki.edu.pl (T.W.); 5 Department of Computer Science and Engineering, Netaji Subhash Engineering College, Kolkata 700152, India; piyali.gini@gmail.com

**Keywords:** S-palmitoylation, post-translational modifications, feature selection, genetic algorithm, random-forest, consensus, knock-out, amino acid index, propensity, synaptic protein

## Abstract

S-palmitoylation is a reversible covalent post-translational modification of cysteine thiol side chain by palmitic acid. S-palmitoylation plays a critical role in a variety of biological processes and is engaged in several human diseases. Therefore, identifying specific sites of this modification is crucial for understanding their functional consequences in physiology and pathology. We present a random forest (RF) classifier-based consensus strategy (RFCM-PALM) for predicting the palmitoylated cysteine sites on synaptic proteins from male/female mouse data. To design the prediction model, we have introduced a heuristic strategy for selection of the optimum set of physicochemical features from the AAIndex dataset using (a) K-Best (KB) features, (b) genetic algorithm (GA), and (c) a union (UN) of KB and GA based features. Furthermore, decisions from best-trained models of the KB, GA, and UN-based classifiers are combined by designing a three-star quality consensus strategy to further refine and enhance the scores of the individual models. The experiment is carried out on three categorized synaptic protein datasets of a male mouse, female mouse, and combined (male + female), whereas in each group, weighted data is used as training, and knock-out is used as the hold-out set for performance evaluation and comparison. RFCM-PALM shows ~80% area under curve (AUC) score in all three categories of datasets and achieve 10% average accuracy (male—15%, female—15%, and combined—7%) improvements on the hold-out set compared to the *state-of-the-art* approaches. To summarize, our method with efficient feature selection and novel consensus strategy shows significant performance gains in the prediction of S-palmitoylation sites in mouse datasets.

## 1. Introduction

Brain functions strictly depend on precise regulation of structural and functional synaptic integrity. Among the mechanisms governing synaptic protein functions, post-translational modifications (PTM) [1,2] play a pivotal role. PTMs may influence synaptic protein activity and turnover, localization at the synapse, and signaling cascades [3,4,5,6].

One of the PTMs is protein S-palmitoylation (S-PALM) involving covalent attachment of palmitic acid (C16:0) to cysteine residue(s) via a thioester bond. Recent studies showed that S-palmitoylation can modulate protein localization, stability, activities, and trafficking and play an essential role in various biological processes, including synaptic plasticity [7,8], cell signaling, cellular differentiation [9], and apoptosis [10].

Unlike other fatty acid modifications, S-palmitoylation is a reversible process, tightly regulated by two groups of enzymes: palmitoyl acyltransferases (PATs, palmitoylating enzymes) and palmitoyl thioesterases (depalmitoylating enzyme). It is widely accepted that repeated cycles of palmitoylation/depalmitoylation are critically involved in regulating multiple protein functions. The molecular mechanisms that lie behind site-specific protein S-palmitoylation remain largely unknown. Several human diseases are often associated with the atypical activity of PATs together with changes in the pattern of S-palmitoylation. S-PALM has been implicated in a wide range of human disease states such as cancer [11], Alzheimer’s disease [12], Parkinson’s disease, cardiovascular disease, schizophrenia [13], or major depressive disorder MDD [14]. Therefore, identifying substrates that undergo S-PALM and specific sites of these modifications may provide candidates for targeted therapy.

Twenty-three PATs have been identified in mammalian cells, which mediate the majority of protein S-palmitoylation. One of the known PATs is a zinc finger DHHC domain-containing protein 7 (Zdhhc7, abbreviated ZDHHC7). This enzyme palmitoylates various synaptic proteins involved in the regulation of cellular polarity and proliferation [15,16]. Moreover, Zdhhc7 is responsible for S-palmitoylation of sex steroid receptors such as estrogen and progesterone receptors [16,17,18]. Importantly, *Zdhhc7*^-/-^ mice developed symptoms characteristic of human Bartter syndrome (BS) type IV because ZDHHC7 protein may affect ClC-K-barttin channel activation [19]. Thus, targeting ZDHHC7 activity may offer a potential therapeutic strategy in certain brain pathophysiological states. Most recently, using the mass spectrometry approach, we have identified sex-dependent differences in the S-PALM of synaptic proteins potentially involved in the regulation of membrane excitability and synaptic transmission as well as in the signaling of proteins involved in the structural plasticity of dendritic spines in the mice brain [18]. Our data showed for the first time sex-dependent action of ZDHHC7 acyltransferase. Furthermore, we revealed that different S-PALM proteins control the same biological processes in male and female synapses [18,19].

Several methods have been developed for the identification of S-palmitoylation target proteins. However, site-specific identification of S-palmitoylation is less studied. Large-scale identification of S-palmitoylation sites mainly relies on mass spectrometry-based methods such as PANIMoni developed in our lab [20] or PALMPiscs or ssABE [21]. These methods have been successfully used to identify a large number of S-palmitoylated proteins in different species, such as rats, mice, or humans. For instance, PANIMoni has been used to describe endogenous S-palmitoylation and S-nitrosylation of proteins in the rat brain excitatory synapses at the level of specific single cysteine in a mouse model of depression [20]. In recent years, results of large-scale proteome databases obtained with PANIMoni, PALMPiscs, or ssABE methods were used to develop tools to predict sites of specific S-palmitoylation in other biological complexes. Several machine learning-based algorithms [22,23,24,25] have been developed for predictions of S-palmitoylation sites such as; NBA-PALM [26] and CSS-PALM [25], but their accuracy is uncertain. Therefore, with the growing number of publicly available large-scale proteome databases of the brain and somatic tissues, there is a need for the development of reliable and accurate computational tools to process them.

Considering the growing recognition for the importance of post-translational modifications of proteins in cell physiology, this study aims to develop a computational tool for predicting S-palmitoylation sites using proteomic data obtained by the mass spectrometry-based method PANIMoni [20]. Most recently, we have successfully used this approach to create a detailed ZDHHC subtype-specific and sex-mouse S-palmitoylome [18,19]. Here, we have used this protein database for validation of the computational tool described in this study.

Our tool is focused on a random forest (RF) [27] classifier-based consensus strategy, which can predict the palmitoylated cysteine sites on synaptic proteins of the male/female mouse dataset. Different heuristic selection strategies have been applied on the physicochemical features from the AAIndex feature database [28] along with position-specific amino acid (AA) propensity information, which eventually generates three different sets of features: (a) K-Best (KB) features, (b) genetic algorithm (GA) based features [29], and (c) a union (UN) of K-Best and GA based features. The experiment has been carried out on three categorized synaptic protein datasets originally described in our previous publications [18,19]; *viz.*, male, female, and combined (male + female). In each experimental group, the weighted data is used as the training set, and the knock-out is used as the hold-out test set for performance evaluation and comparison. A novel RF-driven consensus strategy with efficient feature selection shows significant performance in predicting S-palmitoylation sites in mouse data.

## 2. Results

Our method, RFCM-PALM, predicts the S-palmitoylation sites from the primary sequence information of synaptic proteins. In the mouse model experiments, three categories of data, *viz.,* Male, Female, and Combined, and three different feature sets, *viz.,* KB, GA, UN, along with the RF classifier, have been used. The rationale behind the choice of the RF classifier is elaborated in the Supplementary Section S1 and Table S1. Features are extracted from the sequence motifs of variable length, and detailed experiments are conducted to select the optimum length of such sequence motif. A summary of these experiments is discussed in Section 4.3, and detailed results are reported in the Supplementary Table S2. Finally, the proposed approach presents a three-star consensus model for the final classification task. The efficacy of PTM prediction depends heavily on selecting appropriate feature sets, the choice of the classifier, and the underlying evaluation strategy. In this work, GA-based features show better the area under the curve (AUC) score for male, female, and combined datasets. The UN features show promising performances for the female dataset with higher accuracy, whereas KB and GA features achieve the highest accuracy in male and combined datasets, respectively. Finally, we present a three-star consensus approach for the final classification task. The consensus model significantly improved the performance compared to individual feature-specific models. We have further compared the proposed consensus-based approach, RFCM-PALM, with two *state-of-the-art* methods.

### 2.1. Performance Evaluation

**Figure 1:**
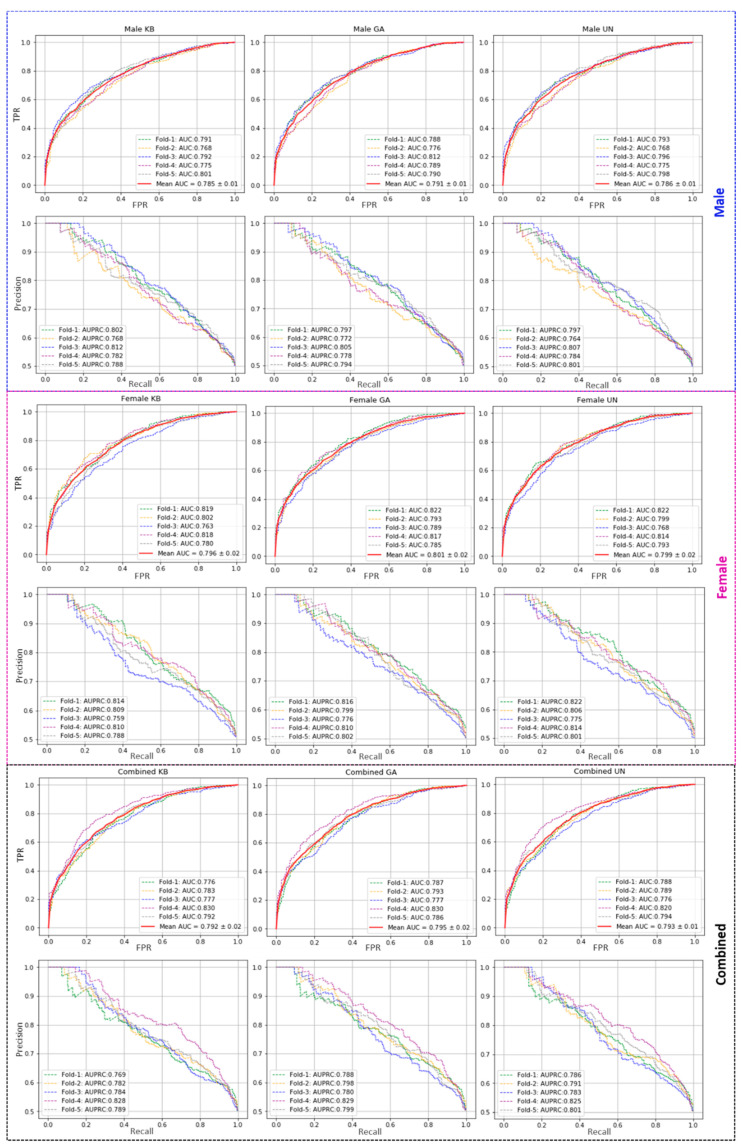
Performance evaluation on three datasets, Male, Female, and Combined. Plots in the 1st, 3rd, and 5th rows show the AUC, and the 2nd, 4th, and 6th rows represent AUPRC, respectively. The 1st, 2nd, and 3rd column-wise plots represent KB, GA, and UN type features-based evaluation.

The performance of the proposed model has been evaluated with five-fold cross-validation on three different feature sets (namely KB, GA, UN) using a RF classifier. Five-fold cross-validation has been introduced to estimate the model’s strength on all three categories of datasets (Male (M), Female (F), and Combined (M + F)), and the performances are reported in Table 1. The individual fold-wise performances on all three datasets are reported in Supplementary Table S3. In all three datasets, the GA-based feature outperforms the rest two in AUC score. However, in our proposed method, for fold-wise testing, the GA-based feature shows a ~79% AUC score for both male and combined datasets, and 80% AUC on the female dataset, surpassing the other two features. For female data, the UN-based feature outperforms KB and GA-based features, having an accuracy score of 71.9% and F1 of 71.3% (see Table 1). The AUC and AUPRC curves from training models are shown in Figure 1.

The knock-out data has been used as the hold-out test set from three categories of data (Male, Female, and Combined) individually. In the knock-out hold-out test set, the GA-based feature shows better performance for all the datasets than other features with an AUC score of ~66.4% in males, 68.6% in females, and 62.5% in combined datasets (please see Table 2). Moreover, GA has higher accuracy in all hold-out test data except the males set, where the KB-based model achieves 62% accuracy. Furthermore, we have introduced a consensus strategy for the final classification of S-PALM on the hold-out test set. Initially, the best models are extracted from the cross-validation strategy for each feature set on the three categories of data set independently.

**Figure 2:**
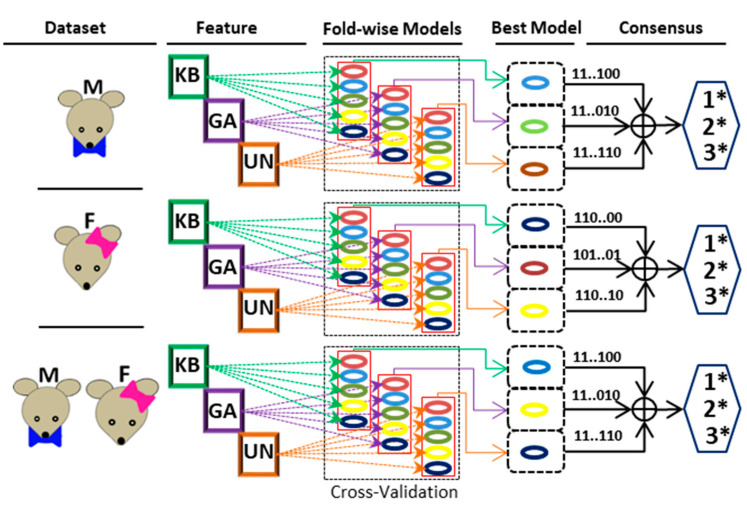
A schematic diagram depicting the underlying consensus strategy for S-PALM prediction.

Thus, the three best models are identified for classification from each data set (Male/Female/Combined). Finally, three consensus-based classifications are obtained for the final classification. The 1*-consensus (1*Con), 2*-consensus (2*Con), and 3*-consensus (3*Con) represent 1, 2, and 3 model confidence scores, respectively. The detailed consensus mechanism is shown in Figure 2, and the results are depicted in Table 2. The 2*Con (2 model confidence) has significantly improved performance compared to the corresponding individual models. Consensus-based performance with different categories of data for hold-out test sets is shown in Table 2.

### 2.2. Comparison with the State-of-the-Art Approaches

To demonstrate the performance of our proposed method, we have compared our approach with existing PTM prediction models. We have identified three *state-of-the-art* approaches for benchmarking purposes, CapsNet [23], MusiteDeep [24,30], and ModPred [31]. The CapsNet [23] is a deep learning-based architecture that provides prediction models for different PTM sites. MusiteDeep [24,30] is a deep-learning-based system that can predict general and kinase-specific phosphorylation sites from primary sequence information. ModPred [31] is a sequence-based PTM prediction tool developed on the structural and functional signatures of proteins. The CapsNet, provides a 10-fold cross-validation result on the benchmark dataset of animal species (metazoa), extracted from the NCBI taxonomy database [32], which has been curated by collecting annotations from Uniprot/Swiss-Prot (August 2007 release) [33] with less than 30% sequence similarity.

Our approach has also been trained with the similar dataset used in CapsNet [23] for S-palmitoylated cysteine prediction for comparison purposes. When compared with all three existing approaches on similar datasets, the performance scores are directly incorporated from Wang et al. [23]. In the proposed model, we have also presented the class-imbalanced learning by imposing a positive-negative ratio at 1:2 along with the balanced learning (1:1). The performance has been compared with the existing approaches concerning the AUC and AUPRC scores (Table 3). Our proposed method outperforms the *state-of-the-art* methods in both metrics. The AUC and AUPRC have improved by 8% in comparison with the earlier best-performing method. Additionally, the proposed approach has surpassed the prior approaches by 32% in the AUPRC score, as depicted in Table 3. The detailed fold-wise evaluation scores are shown in the Supplementary Table S4 (balanced) and Table S5 (imbalanced).

To investigate the significance of our proposed model on a novel S-PALM dataset, we have evaluated and compared the performance with two web servers MusiteDeep [30] and CSS-Palm [25]. MusiteDeep [24,30] is a web resource with a deep-learning framework that can predict and visualize different post-translational modification (PTM) sites from protein sequence information. CSS-Palm [25] is developed based on clustering and scoring strategy (CSS) algorithm and Group-based Prediction System (GPS) algorithm. CSS-Palm is evaluated with two high-performing thresholds, as stated by the authors in [25]. The novel hold-out test data from male, female, and combined sets has been submitted in the above two web servers, and performances have been recorded for comparison purposes (see Table 4). The proposed method has achieved a better result in more balanced metrics F1, and MCC compared to each of these web servers in S-PALM prediction depicting the efficacy of the proposed method on S-PALM prediction. In all three datasets, male, female, and combined, the proposed approach has improved the F1 score by 54%, 52%, and 48%, and MCC score by 7%, 32%, and 13%, respectively.

In this novel hold-out data set, both web servers show high precision (0.827 in MusiteDeep and 0.857 in CSS-Palm) and very low recall (0.0882 in MusiteDeep and 0.1324 in CSS-Palm). A high precision score depicts low false positivity, and low recall depicts the increase in false-negative data, which can be interpreted as a failure for predicting the positive data. This may lead to a biased classification. Low recall also results in a low F1 score, which is the harmonic mean of precision and recall. Not only the recall score, but the MCC score for both the web servers are low, which depicts the failure of the class imbalance issue [34]. In contrast, our proposed method achieves 0.638 precision, and 0.583 recall scores on this hold-out dataset, which shows a more balanced scenario of classification outcome. In addition, our proposed method shows the highest accuracy for all three categories of the data, which outperforms the other two (accuracy improvement by 9%, 15%, and 7% in male, female, and the combined dataset).

## 3. Discussion

Our method, RFCM-PALM, computationally predicts the S-palmitoylation sites using the primary sequence information of the synaptic group of proteins from three categories of mouse data, designed as sex-dependent (male, female) and sex-independent (combined) mode. The computational model has been developed through a rigorous feature selection strategy and optimal model selection for predicting the S-PALM modification sites in a given subsequence window. The proposed model has been evaluated with five-fold cross-validation, and model performances have been compared with the *state-of-the-art* approaches using three different feature sets; KB, GA, and UN. Finally, a consensus strategy is designed based on the feature-specific best models from their cross-validated models. The performance of the consensus model improved significantly compared to *state-of-the-art* approaches. The significant performance improvement in predicting S-PALM modification sites portrayed the efficacy of the proposed method.

The performance of the method may further be enhanced by incorporating deep-learning models. However, the major bottleneck lies with the limitation of adequate training samples. Furthermore, due to the complex nature of the biological experiments, scalability of the experimentally validated samples may not be easy. The development of the RFCM-PALM web server is also in our plans. We also plan to extend the method for other PTM types to predict protein nitrosylation sites in the synaptic proteins.

## 4. Materials and Methods

### 4.1. Dataset Preparation

Experimental S-Palmitoylated datasets are categorized into three groups, male, female, and combined (includes both male and female), where each category contains two types of data: weight (WT) and knock-out (KO). Weight data is used for training, and knock-out data is considered for testing. The dataset was derived using the mass spectrometry-based PANIMoni method from WT and koZDHHC7 mouse brains. The mass spectrometry proteomics data have been deposited to the ProteomeXchange Consortium via the PRIDE partner repository with the dataset identifier PXD025286.

The benchmark dataset for this experiment is constructed with the data available in the said repository. In this experiment, all three benchmarking datasets, namely, male, female, and combined, weight data is considered a train set, and knock-out data is considered the test set for classification. Both male and female datasets contain peptides, modified sites, and assigned proteins. All the modified cysteines are labeled. The cysteines which are labeled with Carbamidomethyl are palmitoylated and are considered as positive data. The cysteines which are labeled as N-ethylmaleimide are not palmitoylated and they constitute the negative data. In this approach, to retrieve the high-quality negative samples, the cysteine positions, which are not within the selected fragments of positive samples, are considered. However, the cysteine position that belongs to the same protein but not in the selected fragment is considered as the negative data for the classification. The cysteine positions with both Carbamidomethyl and N-ethylmaleimide modification create ambiguity in S-PALM identification and thus are discarded from this experiment. The number of positive and negative sites for S-PALM prediction is given in Table 5. In all experiments, the positive and negative ratio is kept as 1:1 for balanced classification. The details of the three benchmark datasets are shown in Table 5.

### 4.2. Features

In this work, we have incorporated amino acid physicochemical properties to design the features for the classification task [28]. The position-specific amino acid propensity is computed from the primary sequence of proteins using the physicochemical properties of each amino acid. We have extracted a λ-length sequence window for each cysteine site with the cysteine at the center of the subsequence.

#### 4.2.1. Position-Specific Amino Acid Propensity (PSAAP)

The position-specific feature of amino acid is introduced for feature design. First, the position-specific amino acid composition is computed for all λ-length sub-sequences in the positive dataset (say, *P_D_*). Initially, the positive data set is divided into five different non-overlapping subsets. For any subset of positive data, the amino acid composition for i−th position is defined as,$\;{\left(A_{1,i}^{P},\;A_{2,i}^{P},\;A_{3,i}^{P},\;A_{4,i}^{P}\ldots \ldots \;A_{20,i}^{P}\right)}^{T}$ where, i=1,2, 3, …λ and 20 amino acids are ordered alphabetically according to their single letter code. Then, the position-specific amino acid composition is computed as the position-wise average over all five subsets, denoted as $\;{\bar{A}}_{1,i}^{P}$. Similarly, the negative dataset is partitioned into five equal partitions where each subset size = $\left|N_{D}\right|=\left|P_{D}\right|$. The position-wise amino acid composition is computed for all negative subsets (as done in the case of $P_{D}$). The position-wise amino acid composition for individual negative subsets is calculated as, ${\left(A_{1,i}^{N},\;A_{2,i}^{N},\;A_{3,i}^{N},\;A_{4,i}^{N}\ldots \ldots \;A_{20,i}^{N}\right)}^{T}$ where, i=1,2, 3, …λ. The average of amino acid composition over five negative subsets is represented as $\;{\bar{A}}_{1,i}^{N}$.

Finally, the propensity of the j−th amino acid at position i in the cysteine sites is computed as:

$$\chi_{i,j}=\frac{\;{\bar{A}}_{j,i}^{P}-{\bar{A}}_{j,i}^{N}}{\;{\bar{\sigma }}_{j,i}^{N}},\;$$

where, $\bar{\sigma }$ represents the standard deviation of j−th amino acid at position i overall negative subsets. With these propensity values, final propensity matrix $ProP_{20\times \lambda }$ is constructed as

$$ProP_{20\times \lambda }=\left[\begin{array}{ccc} \chi_{1,1} & \cdots & \chi_{1,\lambda }\\ \vdots & \ddots & \vdots \\ \chi_{20,1} & \cdots & \chi_{20,\lambda } \end{array}\right]$$

#### 4.2.2. Physicochemical Properties Based PSAAP

In the next level, a physicochemical property-based feature is generated by incorporating the PSAAP (ProP). Currently, there are 566 physicochemical features in the AAIndex database [28]. A numeric score is assigned to each amino acid in the AAIndex database representing any particular physicochemical property scale. Then, the scores are normalized by [0, 1] for all amino acids for individual AAIndex using max–min normalization. From any target subsequence (length=λ), the final feature for any amino acid θ at position ι is for amino acid property φ defined as

$$\tau \left(\theta ,\iota \right)=ProP\left(Ordx\left(\theta \right),\iota \right)\times PHY_{\varphi }\left(\theta ,\iota \right)\;$$

where, Ordx(θ) represent the ordering index of amino acid  θ in ProP matrix and $PHY_{\varphi }\left(\theta ,\iota \right)$.

### 4.3. Sub-Sequence Length Selection

To prepare the dataset, protein sequences are segmented into equal-length windows containing the cysteine at the center position. Amino acid sequences before and after the cysteine position in the sequence window are referred to as backward (BW) and forward (FW) subsequences, respectively. The window size (λ) is varied from 31 to 41 (i.e., |BW|=|FW|=n is varied from 5 to 20 and λ=(2×n+1)). Different length-wise experimental analysis has been carried out to find the optimal subsequence length (window size). Based on the AUC score, it has been found that the performance is optimum when n=19 (window size =2×19+1) as depicted in Table 6. Thus, the length of the subsequence in this approach is set to 19 for all consecutive experiments.

### 4.4. Feature Selection

In the present work, we have introduced two different types of feature optimization strategies for predicting the S-palmitoylation sites in mouse protein. The method includes a K-Best (KB)-based feature optimization strategy and a genetic algorithm (GA)-based feature optimization strategy. We have employed both strategies on three types of datasets (discussed above) and recorded their performances, evaluated on the cross-validated test set, and hold-out test set. A detailed discussion of each feature optimization strategy is discussed in the following section.

#### 4.4.1. K-Best Feature Selection

We have introduced the K-Best feature selection strategy to identify significant and non-redundant features from 566 physicochemical property-based PSAAP features. Initially, individual physicochemical property-wise performance has been evaluated with different varying subsequence lengths (31 to 41). Based on these performances (AUC score), physicochemical properties are sorted/ranked for individual subsequence length. Top-performing K features are extracted from each subsequences length-wise evaluation with four different thresholds of K (as top  25, 50, 75,  and  100). Finally, two sets of features are constructed by considering the intersection of K-best (IB-K) and union of K-best (UB-K) features from different length-wise evaluations.

**Figure 3:**
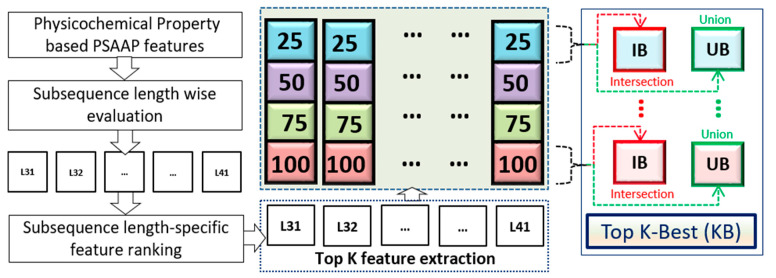
A detailed flow chart for K-Best feature selection.

Once retrieving these K-best feature sets, performance has been evaluated with the merged feature where individual features are concatenated into a single feature vector for final representation. The concatenated feature is generated for the window length 39 (=2 * *n* + 1, where *n* = 19) as it shows superior performance compared to other window lengths. The Union and Intersection-based performance evaluation with four different thresholds (25, 50, 75,  and  100) are depicted in Table 7. Based on AUC and accuracy scores, we concluded that at window length 39 with IB25 gives the best result with the highest AUC score among all (see Table 7), thus constitute the K-best features (KB). Figure 3 shows the detailed workflow for selecting the K-Best feature from the 566 feature set. Finally, the KB feature results in 19, 20 and 21 features in male, female, and the combined datasets, respectively.

#### 4.4.2. Genetic Algorithm Based Feature Selection

Genetic algorithm (GA), which is inspired by the natural selection and evolution process, is a guided random optimized search technique that results in an excellent semi-optimal solution to the feature selection problem [35]. Under GA, fitter children (chromosome) populated from the earlier generation (parents) have a better chance of survival. The feature subsets are encoded as chromosomes are considered as individual and the collection of such chromosomes represent the population. Here, the chromosomes are encoded as a binary string where ‘1’ at any position *i* of represents the selection of *i*-*th* feature and ‘0’ represents the refusal. Each chromosome representing a subset of features is given a fitness score, which is obtained as the AUC in predicting the correct S-PALM modification using this feature subset and RF classifier.

Initially, the 566 physicochemical properties are hierarchically clustered based on the amino acid properties. Then, the hierarchical cluster tree is partitioned into 331 non-singleton and 185 singleton clusters using the same splitting strategy proposed in [36]. In this experiment, GA has used in two steps:

- First, GA is employed over the non-singleton clusters to obtain the best performing feature among the cluster members.
- Second, GA is applied with the newly identified features from the non-singleton clusters and with the remaining features from singleton clusters.

**Figure 4:**
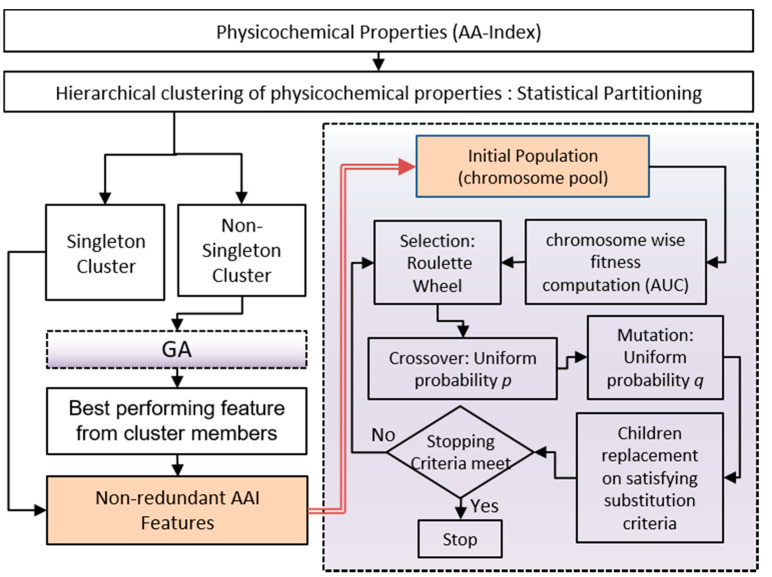
Detailed workflow of GA based feature selection.

In our proposed method, RF is used for classification purposes while evaluating the performance of feature(s) at each generation. However, the AUC score is incorporated in fitness/objective computation. In this experiment, roulette wheel selection strategy and uniform crossover are employed. The crossover probability (p) and uniform mutation probability (q) is set to 0.7 and 0.01, respectively, to populate the next generation chromosome. The positive and negative data ratio is kept as 1:1 for evaluation purposes. The tie between equally performing chromosomes, the one with the lesser number of features, is retained. The method results in the globally best chromosomes. Finally, the GA based approach identified 6 features in male, 7 in female and 21 features in the combined dataset, respectively, for final classification. The overall workflow of GA-based feature design is detailed in Figure 4.

In a nutshell, our tool RFCM-PALM has been developed with effective feature selection and consensus strategy for in silico prediction of S-palmitoylation in mouse protein and shows significant improvement. Sample datasets, supplementary files, and the prediction tool are available at https://github.com/anupgth/RFCM-PALM (accessed on 10 September 2021).

## Figures and Tables

**Table 1:** Performance evaluation of S-palmitoylation prediction from 5-fold cross-validation using three different sets of features on three data types, Male, Female, and Combined.

Type	Feature	5-Fold Cross-Validation					
		AvgAUC	MaxAUC	Precision	Recall	Accuracy	F1
Male	KB	0.785 ± 0.013	0.801	0.732 ± 0.02	0.666 ± 0.02	0.711 ± 0.01	0.697 ± 0.016
	GA	0.790 ± 0.013	0.812	0.726 ± 0.02	0.675 ± 0.02	0.710 ± 0.02	0.700 ± 0.02
	UN	0.786 ± 0.013	0.798	0.726 ± 0.02	0.662 ± 0.01	0.706 ± 0.01	0.693 ± 0.01
Female	KB	0.796 ± 0.02	0.82	0.715 ± 0.02	0.701 ± 0.02	0.708 ± 0.02	0.706 ± 0.02
	GA	0.801 ± 0.018	0.827	0.732 ± 0.02	0.69 ± 0.04	0.718 ± 0.02	0.709 ± 0.02
	UN	0799 ± 0.018	0.821	0.729 ± 0.02	0.698 ± 0.03	0.719 ± 0.02	0.713 ± 0.02
Combined	KB	0.791 ± 0.02	0.830	0.718 ± 0.04	0.689 ± 0.02	0.708 ± 0.03	0.703 ± 0.03
	GA	0.795 ± 0.02	0.830	0.733 ± 0.03	0.684 ± 0.03	0.717 ± 0.02	0.707 ± 0.03
	UN	0.793 ± 0.02	0.820	0.734 ± 0.02	0.670 ± 0.01	0.714 ± 0.02	0.701 ± 0.02

**Table 2:** Performance evaluation using fold-wise and consensus strategy on hold-out test data.

Dataset		Feature	Precision	Recall	Accuracy	F1	MCC	AUC
Male	Fold-wise	KB	0.643 ± 0.01	0.54 ± 0.02	0.620 ± 0.01	0.587 ± 0.01	0.244 ± 0.02	0.661 ± 0.01
		GA	0.629 ± 0.01	0.535 ± 0.02	0.609 ± 0.01	0.578 ± 0.01	0.222 ± 0.02	0.664 ± 0.01
		UN	0.634 ± 0.02	0.532 ± 0.01	0.612 ± 0.01	0.579 ± 0.01	0.227 ± 0.03	0.661 ± 0.01
	Consensus	1*Con	0.585	0.812	0.618	0.68	0.255	0.639
		2*Con	0.667	0.713	0.678	0.689	0.357	0.679
		3*Con	0.676	0.423	0.610	0.520	0.238	0.628
Female	Fold-wise	KB	0.617 ± 0.01	0.566 ± 0.01	0.608 ± 0.01	0.591 ± 0.01	0.216 ± 0.02	0.667 ± 0.01
		GA	0.641 ± 0.01	0.600 ± 0.01	0.632 ± 0.01	0.62 ± 0.01	0.265 ± 0.01	0.686 ± 0.004
		UN	0.622 ± 0.01	0.566 ± 0.02	0.611 ± 0.01	0.593 ± 0.01	0.223 ± 0.02	0.684 ± 0.004
	Consensus	1*Con	0.593	0.792	0.624	0.678	0.264	0.64
		2*Con	0.799	0.706	0.764	0.749	0.532	0.768
		3*Con	0.800	0.447	0.668	0.573	0.373	0.708
Combined	Fold-wise	KB	0.586 ± 0.02	0.475 ± 0.01	0.57 ± 0.01	0.525 ± 0.01	0.142 ± 0.02	0.597 ± 0.01
		GA	0.608 ± 0.02	0.486 ± 0.02	0.586 ± 0.02	0.54 ± 0.02	0.176 ± 0.03	0.625 ± 0.01
		UN	0.605 ± 0.02	0.472 ± 0.02	0.581 ± 0.02	0.53 ± 0.02	0.167 ± 0.03	0.615 ± 0.01
	Consensus	1*Con	0.654	0.719	0.669	0.685	0.340	0.671
		2*Con	0.679	0.669	0.676	0.674	0.353	0.676
		3*Con	0.612	0.374	0.568	0.464	0.148	0.580

**Table 3:** Performance comparison with the *state-of-the-art* methods for S-PALM prediction.

Methods	AUC	AUPRC	Accuracy	F1	MCC
CapsNet [23]	0.780 ± 0.02	0.500 ± 0.07	NA	NA	NA
MusiteDeep [24]	0.771 ± 0.02	0.484 ± 0.05	NA	NA	NA
ModPred [31]	0.8553 ± 0.01	0.5973 ± 0.04	NA	NA	NA
Proposed Method (1:1)	0.936 ± 0.01	0.889 ± 0.02	0.824 ± 0.03	0.799 ± 0.04	0.669 ± 0.05
Proposed Method (1:2)	0.928 ± 0.02	0.785 ± 0.04	0.816 ± 0.02	0.645 ± 0.06	0.577 ± 0.06

**Table 4:** Performance comparison with MusiteDeep [24,30] and CSS-Palm [25] web server with holdout dataset.

Method		Type of Data	Precision	Recall	Accuracy	F1	MCC
MusiteDeep [30]		Male	0.827	0.088	0.535	0.159	0.155
		Female	0.808	0.107	0.51	0.188	0.151
		Combined	0.555	0.0719	0.507	0.127	0.029
CSS-Palm [25]	High Threshold	Male	0.857	0.132	0.555	0.229	0.206
		Female	0.783	0.147	0.524	0.247	0.168
		Combined	0.75	0.129	0.543	0.22	0.153
	Medium Threshold	Male	0.768	0.158	0.555	0.262	0.182
		Female	0.761	0.177	0.532	0.288	0.173
		Combined	0.735	0.179	0.557	0.289	0.176
Proposed Method		Male	0.628	0.539	0.609	0.58	0.222
		Female	0.639	0.583	0.627	0.61	0.254
		Combined	0.623	0.504	0.599	0.556	0.202

**Table 5:** Dataset details of positive and negative sites for all three benchmark data; Male, Female, and Combined.

Category	Type	# Protein	# Cysteine Sites
Male	Positive (*P_D_*)	1077	1870 (Experimental)
	Negative (*N_D_*)	1175	9279 (Identified)
Female	Positive (*P_D_*)	1036	1773 (Experimental)
	Negative (*N_D_*)	1131	8934 (Identified)
Combined (Male + Female)	Positive (*P_D_*)	1180	2083 (Experimental)
	Negative (*N_D_*)	1293	10,403 (Identified)

**Table 6:** Performance with different length of sub-sequences.

Length ( n )	Precision	Recall	Accuracy	F1	AUC
15	0.657	0.792	0.69	0.718	0.765
16	0.701	0.731	0.709	0.715	0.781
17	0.699	0.722	0.706	0.71	0.777
18	0.72	0.731	0.722	0.725	0.788
19	0.724	0.717	0.723	0.72	0.79
20	0.715	0.731	0.719	0.723	0.789

**Table 7:** Performance of top K features.

Feature	Precision	Recall	Accuracy	F1	AUC
IB25	0.724	0.717	0.722	0.72	0.79
IB50	0.715	0.713	0.715	0.714	0.784
IB75	0.702	0.673	0.694	0.687	0.772
IB100	0.707	0.702	0.705	0.704	0.775
UB25	0.72	0.722	0.72	0.721	0.789
UB50	0.714	0.715	0.714	0.714	0.782
UB75	0.709	0.706	0.708	0.707	0.778
UB100	0.703	0.700	0.702	0.701	0.771

## Data Availability

The mass spectrometry proteomics data have been deposited to the ProteomeXchange Consortium via the PRIDE partner repository with the dataset identifier PXD025286.

## Supplementary Materials

Supplementary materials can be found at https://www.mdpi.com/article/10.3390/ijms22189901/s1.

## Abbreviations

GA	Genetic Algorithm
IB	Intersection Based
UB	Union Based
KB	K-Best
RF	Random Forrest
COMBI	Combined
SD	Standard Deviation
CV	Cross Validation
AAI	Amino Acid Index
AUC	Area under the curve
AUPRC	Area under the precision-recall curve
MCC	Matthews Correlation Coefficient
