# Supplementary material for: RFCM-PALM: In-Silico Prediction of S-Palmitoylation Sites in the Synaptic Proteins for Male/Female Mouse Data
Source: Int J Mol Sci. 2021 Sep 14;22(18):9901. doi: 10.3390/ijms22189901 (PMC8467992; doi:10.3390/ijms22189901)
Supplement: Supplementary file 1 [file ijms-22-09901-s001.zip › ijms-1367878-supplementary.pdf]

RFCM-PALM: *In-silico* prediction of S-palmitoylation sites in the synaptic proteins for Male/Female mouse data

Soumyendu Sekhar Bandyopadhyay [1,2,#], Anup Kumar Halder [1,3,#], Monika Zaręba-Kozioł[4], Anna Bartkowiak-Kaczmarek[4], Aviinandaan Dutta[1], Piyali Chatterjee[5], Mita Nasipuri[1], Tomasz Wójtowicz[4], Jakub Wlodarczyk [4,*] and Subhadip Basu[1,*]

1 Department of Computer Science and Engineering, Jadvapur University, Kolkata 700032, India; soumyabane@gmail.com (S.S.B.); anup21.halder@gmail.com (A.K.H.); aviinandaandutta@gmail.com (A.D.); mitanasipuri@gmail.com (M.N.);

2 Department of Computer Science and Engineering, School of Engineering and Technology, Adamas University, Barasat, Kolkata 700126, West Bengal, India;

3 Department of Computer Science and Engineering, University of Engineering & Management, Kolkata 700156, India;

4 The Nencki Institute of Experimental Biology, Polish Academy of Sciences, 3 Pasteur Street, 02-093 Warsaw, Poland; m.zareba-koziol@nencki.edu.pl (M.Z.-K.); a.bartkowiak@nencki.edu.pl (A.B.-K.); t.wojtowicz@nencki.edu.pl (T.W.); j.wlodarczyk@nencki.edu.pl (J.W.);

5 Department of Computer Science and Engineering, Netaji Subhash Engineering College, Kolkata-700152, India; piyali.gini@gmail.com (P.C.);

* Correspondence: bsubhadip@gmail.com (S.B.); j.wlodarczyk@nencki.edu.pl

# Equal Contribution, both shared the first authorship

Received: date; Accepted: date; Published: date

Supplementary Material

S1. Classifier Selection

To check the efficacy of the classifier, the PTM prediction method has been evaluated on a subset of the dataset using KB feature (as described in manuscript) with two machine learning algorithm (SVM [1]and RF [2]). The performance of both classifiers is presented in Table S1. Based on the AUC, F1 and Accuracy scores, RF outperforms SVM. Thus, all the experiments presented in the main manuscript are carried out using RF on all three different types of mouse data (Male, Female, and Combined). For each fold, the positive and negative samples are selected in equal ratio for both train and test sets to obtain unbiased classification.

Table S1. Performance evaluation on SVM and RF classifier.

Classifier	Precision	Recall	Accuracy	AUC	F1
**SVM**	0.671±0.03	0.620±0.08	0.652±0.01	0.720±0.01	0.636±0.03
**RF**	0.669±0.08	0.650±0.02	**0.664**±0.01	**0.728**±0.01	**0.659**±0.01

Table S2. Detailed performance of K-Best features on different length of sub-sequences.

Length	Intersection-based Feature set						Union-based Feature set					
	**IB**	**Precision**	**Recall**	**Accu**	**F1**	**AUC**	**UB**	**Precision**	**Recall**	**Accu**	**F1**	**AUC**
15	25	0.657	0.792	0.69	0.718	0.765	25	0.684	0.719	0.694	0.701	0.77
	50	0.69	0.718	0.698	0.703	0.766	50	0.686	0.724	0.696	0.704	0.764
	75	0.687	0.715	0.694	0.7	0.762	75	0.684	0.713	0.692	0.698	0.761
	100	0.681	0.709	0.689	0.695	0.757	100	0.683	0.698	0.686	0.69	0.758
16	25	0.701	0.731	0.709	0.715	0.781	25	0.699	0.724	0.706	0.711	0.78
	50	0.692	0.724	0.701	0.707	0.773	50	0.7	0.716	0.705	0.708	0.776
	75	0.697	0.716	0.702	0.706	0.772	75	0.689	0.723	0.699	0.705	0.769
	100	0.679	0.709	0.686	0.693	0.763	100	0.694	0.703	0.697	0.698	0.768
17	25	0.699	0.722	0.706	0.71	0.777	25	0.699	0.721	0.706	0.71	0.777
	50	0.698	0.713	0.702	0.705	0.772	50	0.696	0.715	0.702	0.705	0.77
	75	0.684	0.722	0.694	0.702	0.766	75	0.692	0.706	0.695	0.698	0.767
	100	0.688	0.706	0.693	0.697	0.766	100	0.685	0.712	0.693	0.698	0.763
18	25	0.72	0.731	0.723	0.725	0.788	25	0.717	0.733	0.722	0.724	0.787
	50	0.711	0.728	0.716	0.719	0.781	50	0.709	0.726	0.714	0.717	0.78
	75	0.704	0.725	0.71	0.714	0.774	75	0.709	0.728	0.715	0.718	0.777
	100	0.71	0.724	0.714	0.717	0.774	100	0.707	0.72	0.711	0.713	0.773
19	25	0.724	0.717	0.722	0.72	**0.79**	25	0.72	0.722	0.72	0.721	**0.789**
	50	0.715	0.713	0.715	0.714	0.784	50	0.714	0.715	0.714	0.714	0.782
	75	0.702	0.673	0.694	0.687	0.772	75	0.709	0.706	0.708	0.707	0.778
	100	0.707	0.702	0.705	0.704	0.775	100	0.703	0.7	0.702	0.701	0.771
20	25	0.715	0.731	0.719	0.723	0.789	25	0.716	0.73	0.72	0.722	0.787
	50	0.709	0.728	0.715	0.718	0.783	50	0.712	0.728	0.717	0.72	0.782
	75	0.703	0.719	0.708	0.711	0.776	75	0.704	0.719	0.708	0.711	0.778
	100	0.707	0.719	0.71	0.712	0.775	100	0.702	0.713	0.705	0.707	0.775

Table S3. Performance evaluation of 5-fold cross-validation on *Male, Female* and *Combined* dataset with KB, GA and UN features.

Dataset	Feature	Fold	Precision	Recall	Accuracy	AUC	F1	MCC
Male	KB	0	0.748	0.65	0.715	0.791	0.695	0.434
		1	0.717	0.644	0.695	0.768	0.679	0.392
		2	0.736	0.684	0.719	0.792	0.709	0.44
		3	0.699	0.671	0.691	0.775	0.685	0.383
		4	0.761	0.682	0.734	0.801	0.719	0.47
	GA	0	0.714	0.668	0.701	0.788	0.691	0.402
		1	0.702	0.65	0.687	0.776	0.675	0.375
		2	0.762	0.701	0.741	0.812	0.73	0.483
		3	0.714	0.687	0.706	0.789	0.7	0.412
		4	0.74	0.668	0.717	0.79	0.702	0.435
	UN	0	0.734	0.658	0.71	0.793	0.694	0.422
		1	0.716	0.639	0.693	0.768	0.675	0.387
		2	0.735	0.674	0.715	0.796	0.703	0.432
		3	0.701	0.663	0.69	0.775	0.681	0.38
		4	0.747	0.679	0.725	0.798	0.711	0.451
Female	KB	0	0.725	0.723	0.725	0.819	0.724	0.449
		1	0.718	0.698	0.712	0.802	0.708	0.424
		2	0.676	0.715	0.686	0.763	0.695	0.373
		3	0.74	0.709	0.73	0.818	0.724	0.461
		4	0.698	0.658	0.686	0.78	0.677	0.373
	GA	0	0.742	0.684	0.723	0.822	0.712	0.448
		1	0.718	0.698	0.712	0.793	0.708	0.424
		2	0.712	0.746	0.722	0.789	0.728	0.444
		3	0.762	0.686	0.736	0.817	0.722	0.474
		4	0.727	0.638	0.699	0.785	0.68	0.401
	UN	0	0.744	0.723	0.737	0.822	0.734	0.475
		1	0.713	0.675	0.702	0.799	0.694	0.405
		2	0.697	0.729	0.706	0.768	0.713	0.413
		3	0.755	0.698	0.736	0.814	0.725	0.473
		4	0.738	0.667	0.715	0.793	0.7	0.431
Combined	KB	0	0.708	0.671	0.697	0.776	0.689	0.395
		1	0.691	0.695	0.692	0.783	0.693	0.385
		2	0.673	0.688	0.677	0.777	0.68	0.353
		3	0.774	0.726	0.757	0.83	0.749	0.515
		4	0.747	0.668	0.721	0.792	0.706	0.445
	GA	0	0.724	0.673	0.708	0.787	0.697	0.417
		1	0.731	0.659	0.708	0.793	0.693	0.418
		2	0.699	0.692	0.697	0.777	0.696	0.394
		3	0.779	0.731	0.762	0.83	0.754	0.525
		4	0.731	0.666	0.71	0.786	0.697	0.422
	UN	0	0.727	0.685	0.714	0.788	0.705	0.429
		1	0.722	0.656	0.702	0.789	0.688	0.406
		2	0.709	0.656	0.694	0.776	0.682	0.388
		3	0.763	0.688	0.737	0.82	0.723	0.476
		4	0.753	0.668	0.725	0.794	0.708	0.452

Table S4. Performance evaluation of proposed methods for S-palm prediction on the dataset given in [3] with balanced learning (Positive: Negative=1:1).

Fold	Precision	Recall	Accuracy	AUC	AUPRC	F1	MCC
1	0.922	0.714	0.827	0.94	0.89	0.671	0.805
2	0.897	0.696	0.808	0.92	0.872	0.632	0.784
3	0.934	0.749	0.848	0.941	0.904	0.71	0.831
4	0.963	0.678	0.826	0.934	0.901	0.682	0.796
5	0.954	0.649	0.809	0.945	0.889	0.652	0.772
6	0.942	0.649	0.804	0.942	0.883	0.641	0.768
7	0.945	0.828	0.89	0.953	0.929	0.786	0.883
8	0.909	0.633	0.785	0.917	0.863	0.598	0.746
9	0.893	0.726	0.819	0.927	0.878	0.65	0.801
10	0.924	0.708	0.825	0.945	0.889	0.668	0.802

Table S5. Performance evaluation of proposed methods for S-palm prediction on the dataset given in [3] with class imbalanced learning (Positive: Negative=1:2).

Fold	Precision	Recall	Accuracy	AUC	AUPRC	F1	MCC
1	0.971	0.508	0.746	0.938	0.862	0.667	0.56
2	0.945	0.525	0.747	0.913	0.854	0.675	0.552
3	0.965	0.525	0.753	0.935	0.864	0.68	0.568
4	0.98	0.552	0.77	0.935	0.878	0.706	0.601
5	0.985	0.463	0.728	0.949	0.858	0.63	0.538
6	0.96	0.488	0.734	0.933	0.852	0.647	0.537
7	0.953	0.63	0.8	0.949	0.884	0.759	0.637
8	0.914	0.422	0.691	0.908	0.812	0.578	0.454
9	0.934	0.452	0.71	0.904	0.83	0.61	0.491
10	0.97	0.51	0.747	0.94	0.863	0.668	0.561

References:

1. Guo, Y.; Yu, L.; Wen, Z.; Li, M. Using support vector machine combined with auto covariance to predict protein–protein interactions from protein sequences. *Nucleic Acids Res.* **2008**, *36*, 3025–3030, doi:10.1093/nar/gkn159.

2. Breiman, L. Random forests. *Mach. Learn.* **2001**, *45*, 5–32.

3. Wang, D.; Liang, Y.; Xu, D. Capsule network for protein post-translational modification site prediction. *Bioinformatics* **2019**, *35*, 2386–2394.
